# Privatized healthcare for older adults living with chronic illness: A scoping review protocol for synthesizing the state of knowledge on their experiences

**DOI:** 10.1371/journal.pone.0317184

**Published:** 2025-02-12

**Authors:** Stuart McKinlay, Christine L. Sheppard, Paige Brown, Luxey Sirisegaram, Kristina M. Kokorelias

**Affiliations:** 1 Division of Geriatric Medicine, Department of Medicine, Sinai Health System and University Health Network, Toronto, ON, Canada; 2 Undergraduate Medical Education, Temerty Faculty of Medicine, University of Toronto, Toronto, ON, Canada; 3 Faculty of Social Work, University of Toronto, Toronto, ON, Canada; 4 Department of Occupational Science and Occupational Therapy, Temerty Faculty of Medicine, University of Toronto, Toronto, ON, Canada; 5 Rehabilitation Sciences Institute, Temerty Faculty of Medicine, University of Toronto, Toronto, ON, Canada; Miami Cancer Institute, Baptist Health South Florida, UNITED STATES OF AMERICA

## Abstract

As global populations age, the prevalence of chronic illness among older adults is increasing, intensifying the burden on healthcare systems. Research shows that today’s older adults, especially those over 65, are more likely to suffer from multiple chronic conditions than previous generations. This demographic shift underscores the urgent need for healthcare systems capable of addressing complex, long-term health needs. The rise of privatized healthcare—services provided by non-governmental entities and funded through private insurance or out-of-pocket payments—has become a significant feature of the healthcare landscape, affecting how older adults receive care. In contrast to public healthcare systems, which are government-funded and aim to ensure universal coverage, privatized models often involve substantial private costs. Hybrid systems, such as those in Australia, combine public and private elements to offer comprehensive services. However, even in predominantly public systems like Canada, private costs for non-covered services persist. This scoping review protocol outlines a plan to identify (1) the potential role of privatized healthcare models in countries with public or hybrid healthcare in addressing health needs, (2) challenges and limitations associated with these models for older adults living with chronic conditions, and (3) current gaps in knowledge regarding the impact of privatized healthcare on care outcomes for older adults, based on the available literature.. It aims to explore the experiences and challenges of this population across various countries with public or hybrid healthcare systems. The review will use a structured methodology based on the Arskey and O’Malley guidelines and the Joanna Briggs Institute framework, focusing on qualitative studies published in the past 20 years. By comparing findings across different settings, the review seeks to provide a comprehensive understanding of how privatized healthcare models affect older adults and inform future research and policy development.

## Introduction

As populations age worldwide, the number of older adults living with chronic illness continues to rise [1]. Research indicates that later-born generations of older adults, such as those who are 65 and older today, are more likely to have multiple chronic conditions compared to their predecessors, which exacerbates the strain on healthcare systems and insurance frameworks. [2]. This demographic shift brings into sharp focus the need for healthcare systems capable of addressing complex, long-term health needs. In many countries, privatized health services have become a significant component of the healthcare landscape, impacting how older adults receive care [3]. Privatized healthcare refers to healthcare services provided by non-governmental entities, often funded through private insurance or out-of-pocket payments [4]. This system contrasts with public healthcare, which is funded and managed by government agencies [5].

Public healthcare systems, such as those in the United Kingdom and Canada aim to ensure universal coverage for all citizens [6]. In contrast, private healthcare systems, like those in the United States, rely heavily on private insurance and out-of-pocket payments, with limited government intervention [6,7]. However, many countries, such as Australia, adopt hybrid models that combine elements of both public and private systems to provide a comprehensive range of services [8,9]. Even in predominantly public healthcare systems, such as Canada, there are often private costs associated with healthcare [10]. For instance, while the Canadian public system covers essential medical services, patients may still incur out-of-pocket expenses for prescription medications, dental care, vision care, and other non-covered services [10,11]. Additionally, some Canadians choose to purchase private health insurance to cover these additional costs and to gain access to private healthcare providers for faster or more specialized services [12]. Many employers also include health insurance as part of compensation packages [13]. This blend of public funding and private expenditure highlights the complexity of healthcare financing, even in systems designed to provide universal coverage.

To address the growing pressures within public healthcare systems, there has been a noticeable shift towards privatization and hybrid models [14–18]. For example, online medical clinics, subscription-based models for rapid doctor access, private specialist and screening clinics (such as those for MRIs and X-rays) represent a trend towards expanding private sector involvement in healthcare delivery in counties with public health care [19–21]. These privatization measures are often proposed as solutions to systemic issues like physician shortages and prolonged wait times [16]. However, this shift introduces a complex dynamic that significantly impacts older adults, a population that may experience unique challenges as public systems evolve. The implications of these privatization trends on aging populations need to be carefully examined, as older adults often face distinct healthcare needs and may be disproportionately affected by changes in healthcare accessibility and quality [22,23]. Understanding these impacts is crucial to ensuring that reforms support the needs of all patients, particularly those with chronic conditions, as such diseases often require ongoing, comprehensive care that can be significantly affected by shifts in healthcare delivery models [24].

The rise of privatized healthcare is driven by factors such as increasing healthcare costs [25] and the desire for more efficient and specialized services [25,26]. Studies have found that individuals with private insurance may experience less access to care, higher costs, and decreased satisfaction compared to those with public insurance [27]. Moreover, some research indicates that private sector healthcare systems may have greater risks of low-quality care, particularly in less regulated environments [4]. This includes issues such as unnecessary testing and treatment, and higher risks of complications [4]. Yet, in countries like Canada, debates around privatization highlight the potential benefits of reducing surgical wait times and alleviating pressure on public systems, although concerns about increased inequality and resource diversion persist [16,21]. On the contrary, other scholars suggest that private healthcare systems may struggle to provide the comprehensive, coordinated care that many older adults require, especially those with multiple chronic conditions [28].

Overall, research on the experiences of older adults in privatized healthcare systems reveals a mixed picture. One USA study found that older adults living with multiple chronic conditions who lacked private insurance were more likely to experience depressive symptoms [29]. On the other hand, private insurance and Medicare helped mitigate the risk of cognitive impairment, particularly among non-Hispanic Whites with multiple chronic conditions and among Blacks regardless of the number of chronic conditions [29]. These findings suggest that private insurance can play a crucial role in reducing mental health disparities among individuals coping with multiple chronic conditions [29]. Furthermore, in a 2017 study of 1,593 older adults living in an urban center in Brazil, access to a private health plan was associated with an increase in hospitalization rates, independent of multimorbidity. These findings suggests that private healthcare may be associated with health service use, although further investigation would be required to clarify the details of this potential relationship [6,30,31].

Given the complexities and varied experiences associated with privatized healthcare, there is a critical need to synthesize the state of knowledge on this topic. This scoping review is timely as it seeks to compare the healthcare experiences of older adults living with chronic illness across geographical settings, to identify and explore potential solutions to diverse health system challenges. To the best of our knowledge, this is the first scoping review to explore the perspectives of older adults living with chronic illness on privatized healthcare, in all countries with a public or hybrid system. While previous studies in the field have explored the experiences of older adults using public or private services in designated countries, more research is needed to examine findings across landscapes and identify central themes and considerations. Therefore, the goal of this scoping review is to provide a comprehensive, narrative analysis of published literature in the field, with the intention of informing future research and healthcare decision-making. This scoping review focuses on individuals aged 60 and above, a demographic increasingly impacted by early-onset chronic conditions and healthcare challenges [32], particularly in privatized or hybrid healthcare systems. The Arskey and O’Malley guidelines will provide a structured approach to defining the research question, identifying relevant literature, and determining the scope of the review [33].

## Methods

Scoping reviews aim to comprehensively map and synthesize evidence on a broad topic area, often encompassing diverse study designs and methodologies. Unlike systematic reviews, which prioritize the critical appraisal of individual study quality to assess the reliability and validity of findings, scoping reviews focus on identifying the breadth and depth of existing literature [33].

The Preferred Reporting Items for Systematic Reviews and Meta-Analysis Protocols (PRISMA-P) checklist [34] was used to develop the protocol (S1 File)

Our research will follow the Arskey and O’Malley guidelines [33] and will be further informed by the Joanna Briggs Institute (JBI) framework for scoping reviews [35]. These guidelines emphasize a systematic method for scoping reviews, focusing on the breadth of evidence and mapping out key concepts. In parallel, the JBI framework will offer detailed guidance on the methodological processes, including the development of inclusion and exclusion criteria, data extraction, and synthesis of findings [35]. By merging these frameworks, our research will benefit from a robust methodological structure that combines the broad scope and mapping approach of the Arskey and O’Malley guidelines with the JBI framework’s emphasis on systematic assessment and practical application. Findings will be reported in line with the PRISMA extension for Scoping Reviews (PRISMA-ScR) [36]. We will incorporate de-identified interview material in our Results section to supplement central themes extracted from included studies. Our stepwise approach, in accordance with the scoping review framework, is outlined below.

### Step 1: Research question development

The purpose of the forthcoming scoping review will be to explore the experiences and perspectives of older adults living with chronic illness on privatized healthcare, in countries that have a public or hybrid healthcare model: Albania, Algeria, Argentina, Australia, Austria, Bahrain, Belgium, Bhutan, Botswana, Brazil, Brunei, Canada, China (including Hong Kong, special administrative region of China), Costa Rica, Croatia, Cuba, Cyprus, Czechia, Denmark, Finland, France, Germany, Greece, Iceland, Israel, Italy, Japan, Kuwait, Luxembourg, Macau, Malaysia, Maldives, Mauritius, Morocco, The Netherlands, New Zealand, Norway, The Philippines, Portugal, Saudi Arabia, Serbia, Seychelles, Singapore, Slovenia, South Korea, Spain, Sri Lanka, Sweden, Switzerland, Thailand, Trinidad and Tobago, Turkey, United Arab Emirates, and The United Kingdom [37]. This scoping review will aim to identify: (1) the impact of privatized healthcare models (intervention) on older adults aged 60 and older living with chronic conditions (population) in countries with public or hybrid healthcare systems on addressing health needs(2) the challenges and limitations (outcome) associated with these models for older adults living with chronic conditions, and (3) the gaps in knowledge regarding the effects of privatized healthcare models (intervention) on care outcomes for older adults (population) based on the available literature.

### Step 2: Identifying studies

Our search strategy will be developed internally with members of our research team, and with support from a health science librarian (Sinai Health System). We also will consult two health policy experts from a health policy thinktank in the finalization of our search strategy. We will conduct a keyword launch, in combination with the use of the Medical Subject Headings (MeSH) feature, to ensure a thorough literature search. The literature search will not be limited to a specific timeframe. We will use the ‘AND’ Boolean operator to gather results that combined keywords. See S2 File for a sample search strategy. We will conduct our search strategy in peer-reviewed databases, including PubMed/MEDLINE, CINAHL (Cumulative Index to Nursing and Allied Health Literature), Embase, Scopus, Cochrane Library, ProQuest Health & Medical Collection, Web of Science, PsycINFO, and EconLit. In addition to the primary database search, we will conduct a targeted search on Google Scholar, reviewing the first five pages of search results to ensure that any relevant studies not captured by our database search are included in the review. This will help identify potentially overlooked studies or emerging research. A manual search of the references of included studies, as well as forward citation searching (i.e., to identify newer research that has cited a specific original publication as forward citation searching refers to the process of identifying newer studies that have cited a specific original publication, which helps locate more recent, relevant research), will also be conducted prior to concluding our search [38].

Older adults living with chronic illness, aged 60 years or older, with experiences and perspectives on privatized healthcare will be the population of interest in our scoping review. A publication period of the past 20 years will be supported in our scoping review to extract results based on recent experiences. Only studies set in countries with a public aspect to their healthcare model will be included in our scoping review (i.e., we mean healthcare systems where the government plays a significant role in financing, regulating, or delivering healthcare services). All relevant studies published in or translated to the English language will be sought. Non-English papers will be assessed for relevance based on their abstracts and key content that will be translated using Google Translate [39]. If a non-English paper is identified as potentially relevant, it will be translated into English using a professional translation service. We will use KeyLingo translation services (https://keylingo.com/), which specializes in translating academic research. This service ensures accurate and contextually appropriate translations. Thus, the translation process will involve initially assessing the paper’s relevance through its abstract and key sections, such as the introduction, methods, and conclusions, followed by full translation if necessary. The translated paper will then be reviewed for inclusion in the scoping review based on the same inclusion and exclusion criteria applied to English-language studies.

Grey literature will be excluded due to concerns regarding its methodological reliability [40]. Additionally, incorporating grey literature would significantly increase the volume of data to be reviewed, potentially straining our capacity to thoroughly assess and synthesize the evidence within the constraints of this review.

We anticipate conducting our literature search in November 2024 and completing our study screening and data extraction by February 2025.

### Step 3: Selecting studies

Please refer to Table 1 for a comprehensive summary of inclusion and exclusion criteria.

**Table 1:** Inclusion and exclusion criteria.

Criteria	Inclusion	Exclusion
Population	Older adults (aged 60 and above) living with chronic illness	Individuals under 60 years of age; older adults living without chronic illness. Individuals with acute conditions.
Concept	Experiences and perspectives on privatized healthcare	Studies not focused on privatized healthcare models; studies focusing on general healthcare without specifying privatization. Studies focused on an intervention without qualitative insights.
Context	Countries with public or hybrid healthcare models: Albania, Algeria, Argentina, Australia, Austria, Bahrain, Belgium, Bhutan, Botswana, Brazil, Brunei, Canada, China (including Hong Kong, special administrative region of China), Costa Rica, Croatia, Cuba, Cyprus, Czechia, Denmark, Finland, France, Germany, Greece, Iceland, Israel, Italy, Japan, Kuwait, Luxembourg, Macau, Malaysia, Maldives, Mauritius, Morocco, The Netherlands, New Zealand, Norway, The Philippines, Portugal, Saudi Arabia, Serbia, Seychelles, Singapore, Slovenia, South Korea, Spain, Sri Lanka, Sweden, Switzerland, Thailand, Trinidad and Tobago, Turkey, United Arab Emirates, and The United Kingdom [37]	Countries with fully privatized healthcare systems or without a public healthcare component
Study design	Qualitative methodologies	Quantitative, reviews, case studies, opinion pieces, editorials without empirical data, conference abstracts, non-peer-reviewed sources, doctoral theses
Time periods	Studies published within the last 20 years (2004-2024)	Studies published before 2004
Setting	Community, primary care, and long-term care settings	Acute care settings (unless they include post-acute perspectives relevant to the study population)

We will use EndNote (x21) to identify and remove duplicate studies extracted from the database searches. Studies will then be imported into Covidence software to complete two-step study screening. First, title-abstract (level 1) screening, followed by full-text (level 2) screening, will be conducted independently by two members of the research team. Discrepancies will be resolved with consultation from a third member of the research team (CS or LS). If agreement cannot be reached, a team meeting will be organized to resolve the remaining study screening conflicts. A team meeting will be conducted between level 1 and level 2 screening to clarify inclusion and exclusion criteria and address outstanding questions.

### Step 4: Extracting data

Two members of the research team will independently extract study data using Excel. A data extraction form will be created iteratively by members of the research team, with consultation from members of our advisory committee following full-text review of included studies. Preliminary data to be extracted will include the following: author name, publication year, study jurisdiction, rural/urban, healthcare setting and country, study design, data collection methods, sample size, participant demographics (e.g., sex/gender, age/mean age, chronic condition), intervention/comparator (if applicable and experiences of the intervention), conceptualization/operationalization of private healthcare, experiences and perspectives of the older adult participants, results, and recommendations. To ensure concordance, both researchers will extract data from approximately five of the same studies (or ~ 25% of the final included studies). A meeting will be conducted at this time to discuss discrepancies, and the data extraction will be refined as needed. Discrepancies will once again be resolved with consultation from a third member of the research team (CS or LS). If significant discrepancies in data extraction exist, a full meeting with all members of the research team will be conducted to discuss the data extraction form, prior to completing the remaining data extraction. Once all data is extracted and conflicts are resolved, a senior member of the research team will review the study data for verification. A risk of bias assessment and quality assessment will not be conducted as in line with scoping review methodologies [33]. As this is a scoping review, which aims to map the breadth of existing literature rather than critically appraise individual studies, we have chosen not to conduct a formal risk of bias assessment. The primary goal of this scoping review is to provide an overview of the available evidence, identify gaps in the literature, and inform future research, irrespective of study quality.

### Step 5: Collecting and analyzing data

Data extracted from included studies will be systematically charted into tables, capturing study characteristics, participant demographics, key findings, and relevant details pertaining to privatized healthcare for older adults living with chronic illness. We will format our findings in tables using Microsoft Word. We will analyze the data content in the context of our research question, using a content analysis framework specific to scoping reviews [41]. NVivo 14 software will be used to organize qualitative data [42]. The number of codes and themes will depend on the richness and diversity of the qualitative data collected across the studies. We anticipate generating a comprehensive set of initial codes based on emerging concepts and recurring topics identified during the data coding process. These initial codes will then be organized into broader thematic categories through an iterative process of data review, comparison, and refinement. The final number of thematic categories will reflect the key findings and insights derived from the qualitative data analysis, aiming to capture the complexity and depth of older adults living with chronic conditions’ experiences within privatized healthcare settings. This approach ensures a rigorous and systematic exploration of the qualitative evidence, facilitating a nuanced understanding of the implications and challenges associated with privatized healthcare models in countries with a public or hybrid healthcare system, for this specific demographic.

## Knowledge translation

Our scoping review will employ an integrated knowledge translation (IKT) approach [43], which emphasizes collaboration with key stakeholders throughout the research process. This method enhances the relevance, usability, and applicability of our findings [43]. Specifically, the project team collaborated with knowledge user partners to input shaped the study’s objectives, the inclusion criteria, and the data extraction framework. The knowledge user partners consisted of two think-tanks within academic centers within Ontario, Canada and involved representatives from one public health organization within Canada.

The IKT approach facilitates the co-creation of knowledge by integrating diverse perspectives, ensuring that the review’s outcomes address practical needs [44]. This collaboration will continue during the dissemination phase, where knowledge users will be involved in interpreting findings and identifying strategies to implement them. Such an approach is particularly critical as countries like Canada increasingly incorporate private healthcare elements within publicly funded systems. This focus aligns with our goal of providing actionable insights that improve health outcomes for older adults and inform broader health system reforms.

## Discussion

The prevalence of chronic illnesses among older adults is a growing concern worldwide, particularly in the context of aging populations and the consequent strain on healthcare systems. This scoping review protocol aims to outline the methodology of a forthcoming review that will explore the role, challenges, and limitations of privatized healthcare models in addressing the needs of older adults living with chronic conditions in countries with public or hybrid healthcare systems [24]. By synthesizing the available literature, this review seeks to provide a comprehensive understanding of how privatized healthcare impacts this vulnerable population.

The importance of this review lies in its potential to shed light on the varied experiences of older adults within different healthcare models. The inclusion of countries with public or hybrid systems allows for a comparative analysis, highlighting how privatized elements within these frameworks can influence experiences for older adults. This is particularly relevant as many countries grapple with balancing public and private healthcare provisions to meet increasing healthcare demands. The review will also explore the specific challenges faced by older adults, such as access to care, affordability, quality of care, and the availability of chronic disease services [24]. Lastly, this review will support the consideration of the social determinants of health, such as socio-economic status, which may influence the experiences and outcomes of older adults within privatized healthcare systems. Understanding these factors is essential for developing equitable healthcare policies that do not disproportionately disadvantage certain groups.

The findings of this scoping review will provide critical insights into how privatized healthcare models operate within public or hybrid systems, specifically their ability to address the unique and complex health needs of older adults living with chronic conditions. In countries like Canada, where shifts toward privatized elements are occurring, these insights can guide evidence-informed policymaking by identifying where privatized models may enhance care delivery and where they risk exacerbating inequities. For instance, the review may highlight whether privatized models improve access to timely, specialized care for older adults or create barriers due to cost or fragmentation of services. These findings can also inform targeted strategies for integrating privatized healthcare components to complement public systems effectively, ensuring that care remains accessible, affordable, and equitable for vulnerable populations.

### Limitations

This scoping review has some limitations. Namely, our findings – which aim to explore the experiences of older adults living with chronic illness utilizing privatized healthcare service or services – can be heterogenous and highly variable across setting and context. We will make efforts to mitigate this limitation within our scoping review. Specifically, we will focus on extracting key overarching themes relevant to patient care to ensure our findings may be broadly applied. Ultimately, this approach strives to ensure our findings are generalizable to complex patient populations with unique health needs or values, and comprehensive in synthesis.

## Conclusion

This scoping review protocol outlines a comprehensive approach to examining the experiences and perspectives of older adults living with chronic illness in privatized healthcare settings within countries that predominantly have public or hybrid healthcare systems. By systematically mapping the existing literature, we aim to identify the potential impact of privatized healthcare models in addressing the complex health needs of this population. The findings will provide valuable insights into the current state of knowledge, highlight gaps, and inform future research directions and policy decisions. Our iKT approach, involving collaboration with various stakeholders, ensures that the review will produce relevant and practical knowledge, contributing to a more equitable and effective healthcare landscape for older adults. We anticipate that the results of this review will guide healthcare practitioners, policymakers, and researchers in improving care delivery and addressing disparities in access to and quality of healthcare services.

## Supporting information

S1 File Prisma P. (DOCX)

S2 File Search strategy. (DOCX)
