# Supplementary material for: Privatized healthcare for older adults living with chronic illness: A scoping review protocol for synthesizing the state of knowledge on their experiences
Source: PLoS One. 2025 Feb 12;20(2):e0317184. doi: 10.1371/journal.pone.0317184 (PMC11819467; doi:10.1371/journal.pone.0317184)
Supplement: S2 File — (DOCX) [file pone.0317184.s002.docx]

Supplemental File 2: Key Terms for Search Strategy

("Aged"[MeSH] OR "elderly"[tw] OR "aging"[tw] OR "aged, 80 and over"[MeSH] OR "older adults"[tw] OR "senior citizens"[tw])

AND

("Chronic Disease"[MeSH] OR "Non-communicable Diseases"[MeSH] OR "chronic disease"[tw] OR "chronic illness"[tw] OR "chronic conditions"[tw] OR "long-term conditions"[tw])

AND

("Health Care Reform"[MeSH] OR "Privatization"[MeSH] OR "healthcare privatization"[tw] OR "private healthcare"[tw] OR "privatized health systems"[tw] OR "private sector healthcare"[tw])

AND

("Health Systems"[MeSH] OR "Public Health"[MeSH] OR "public healthcare"[tw] OR "hybrid healthcare"[tw] OR "public-private healthcare"[tw] OR "government funded healthcare"[tw] OR "national health service"[tw] OR "universal healthcare"[tw] OR "publicly funded health systems"[tw])

AND

("Health Services Accessibility"[MeSH] OR "Health Outcomes"[MeSH] OR "healthcare accessibility"[tw] OR "health outcomes"[tw] OR "care outcomes"[tw] OR "care needs"[tw] OR "quality of care"[tw] OR "health service utilization"[tw] OR "equity of care"[tw])
